# Lipidome Complexity in Physiological and Pathological Skin Pigmentation

**DOI:** 10.3390/ijms26146785

**Published:** 2025-07-15

**Authors:** Emanuela Bastonini, Daniela Kovacs, Vittoria Maresca, Monica Ottaviani, Anna Di Nardo, Enrica Flori, Giorgia Cardinali, Stefania Briganti

**Affiliations:** Laboratory of Cutaneous Physiopathology and Integrated Center of Metabolomic Research, San Gallicano Dermatological Institute, IRCCS, 00144 Rome, Italy; emanuela.bastonini@ifo.it (E.B.); daniela.kovacs@ifo.it (D.K.); vittoria.maresca@ifo.it (V.M.); monica.ottaviani@ifo.it (M.O.); anna.dinardo@ifo.it (A.D.N.); stefania.briganti@ifo.it (S.B.)

**Keywords:** lipidomics, lipids, skin, melanogenesis, melanin, melasma, solar lentigo, vitiligo

## Abstract

Skin pigmentation results from complex cellular interactions and is influenced by genetic, environmental, and metabolic factors. Emerging evidence highlights the multiple pathways by which lipids regulate melanogenesis and points to lipid metabolism and signaling as key players in this process. Lipidomics is a high-throughput omics approach that enables detailed characterization of lipid profiles, thus representing a valid tool for evaluating skin lipid functional role in both physiological melanogenesis and pigmentary disorders. The use of lipidomics to gain a deeper comprehension of the role of lipids in skin pigmentation is still an evolving field, but it has allowed the identification of significant lipid dysregulation in several pigmentary pathologies. This review summarizes the current knowledge on the involvement of lipids in skin pigmentation, focusing on lipid profile alterations described in hyper- and hypopigmentary disorders such as post-inflammatory hyperpigmentation, melasma, solar lentigo, and vitiligo. Lipidomic profiling reveals disease-specific alterations supporting the pivotal role of lipid signaling in the physiopathological mechanisms of melanogenesis. These findings provide insights into disease pathogenesis and show promise for the discovery of biomarkers and innovative therapeutic strategies for pigmentary disorders.

## 1. Introduction

In recent years, biological sciences have moved towards studying global, systems-based processes rather than individual molecules. The development of high-throughput omics technologies, such as genomics, epigenomics, transcriptomics, proteomics, metabolomics, and lipidomics, has allowed researchers to characterize biological and pathological processes in a more comprehensive manner [1,2,3,4,5,6]. Lipidomics has gained significant attention across various fields, including medicine. Research into lipid homeostasis and its deregulation in the human body has begun in many medical areas, yielding interesting findings that could be further applied in everyday medical practice [7,8,9,10]. In this view, the cutaneous surface lipidome is increasingly recognized as a significant source of biomarkers [11,12]. This underscores its importance in dermatological research. Lipid imbalances are common in several inflammatory skin diseases and affect both the choice and efficacy of treatments [12,13]. Therefore, analysis of the epidermal lipidome has been proposed as a method to predict the progression of these diseases. However, although lipid signaling and lipid modification of skin cells have been identified as major players in the process of physiological melanogenesis and in the onset of pigmentation disorders, the application of lipidomics for studying the physiopathology of these skin diseases is still a developing field.

In this review, we aim to provide an up-to-date overview of the role of lipid signaling and metabolism in the physiological and pathological mechanisms of melanogenesis, focusing on the use of analytical lipid profiling to understand molecular mechanisms and develop innovative therapeutic interventions for pigmentary disorders.

## 2. Lipidomics Principles

Lipidomics is an emerging field within omic sciences [14,15,16] and focuses on the complex lipid profile in biological systems. It provides comprehensive insights into lipids using analytical chemistry and data mining tools. Lipidomics aims to fully characterize lipid molecular species and their biological roles [17,18].

Lipids are crucial metabolites that play key roles in various functions like structure, proliferation, differentiation, and metabolism of cells, as well as enzyme activation, apoptosis, and signal transduction [7,19]. Lipid molecules are diverse in structure and function, classified as hydrophobic or amphiphilic depending on their chemical properties. Amphiphilic lipids are found in vesicles, membranes, or liposomes in aqueous environments. Biologic lipids are categorized into classes and subclasses based on their head groups and the type of linkage between the head group and aliphatic chains [20,21]. According to the Lipid MAPS classification, there are eight main classes: fatty acyls, glycerolipids, glycerophospholipids, sphingolipids, sterol lipids, prenol lipids, saccharolipids, and polyketides.

Characterizing the lipidome in biological samples is challenging due to the vast diversity of lipids and their complex structures [7,22]. The combination of different fatty acids and head groups creates significant structural variation, making molecular profiling difficult. Additionally, the presence of isobaric lipid species and the detection of minor lipid species add to the complexity of lipidomics.

Advancements in analytical techniques such as mass spectrometry (MS), nuclear magnetic resonance (NMR), and high-performance liquid chromatography (HPLC) have significantly boosted research in lipidomics [23,24,25,26,27]. Depending on the research objectives, lipidomic analyses can be approached in three analytical modes: focused lipidomics (lipid profiling), targeted lipidomics (targeted lipid analysis), and untargeted lipidomics (global lipid profiling) [28,29]. Focused lipidomics analyzes specific lipid metabolites or pathways using tandem mass spectrometry (MS/MS) for its high sensitivity and robustness [30]. Targeted lipidomics, using liquid chromatography-mass spectrometry (LC-MS) with multiple reaction monitoring (MRM) or selected reaction monitoring (SRM), targets specific lipid species and provides high sensitivity, selectivity, and a broad dynamic range [31,32]. Quantification is achieved using isotopically labeled internal standards for accurate calibration [33,34].

The untargeted lipidomics approach focuses on analyzing a broad range of lipids in biological samples, typically generating relative quantitative data for a large number of analytes. However, it primarily targets species of higher abundance due to the limited dynamic range [35,36]. This approach provides a wide array of lipidomic features in a single run, with identification based on retention time and mass traces, using experimental data or MS/MS libraries. High-resolution mass spectrometry (HRMS) platforms, such as quadrupole–time-of-flight (QTOF), Orbitrap, and Fourier transform ion cyclotron resonance (FT-ICR), are essential for accurately determining lipid species and distinguishing them from isobaric compounds [37,38]. To streamline data analysis, a semi-targeted approach can be used post-acquisition, focusing on previously identified compounds.

Lipid species detected by MS-based approaches are typically annotated using dedicated databases like the LIPID MAPS, developed by the LIPID MAPS consortium [39,40]. This database categorizes lipid species by families, classes, and subclasses based on their accurate mass and structure. It is freely accessible and can be downloaded for automatic annotation. LIPID MAPS compiles various other databases, such as LipidBank, LIPIDAT, and LipidBlast [41,42]. To analyze and interpret high-dimensional lipidomics data in a clinical context, advanced biostatistical tools are employed [43,44]. These include supervised and unsupervised methods like partial least squares discriminant analysis (PLS-DA), orthogonal projection–potential structure analysis (OPLS-DA), principal component analysis (PCA), clustering analysis, linear discriminant analysis, and other stoichiometric techniques [45].

The skin undergoes complex lipid metabolism, largely influenced by dietary intake, with lipids essential for forming and maintaining the epidermal barrier, defending against microbes, and supporting membrane structure and cellular functions.

Its primary function is to form a lipid-based barrier that protects the body from external irritants and infections (outside-in barrier) while preventing water loss (inside-out barrier) [46,47,48]. The outermost epidermal layer, the stratum corneum (SC), is composed of flattened, enucleated corneocytes embedded in a lipid-rich extracellular matrix. This matrix includes ceramides (CERs), cholesterol (CH), and free fatty acids (FFAs), which form lamellar membranes in a mildly acidic environment, essential for skin permeability [49,50,51,52]. An equimolar balance of CERs, CH, and FFAs in the SC is crucial for maintaining proper water retention and preventing excessive trans-epidermal water loss (TEWL) and ion permeability [53,54,55].

Within the epidermis, keratinocytes (KCs) undergo regulated proliferation and differentiation, forming four layers: the stratum basale, stratum spinosum, stratum granulosum, and SC, from the inside to the outside. The rapid turnover of KCs requires a precise program of lipid synthesis and distribution, coordinated with terminal differentiation and cell death. As KCs differentiate, their lipid composition changes significantly [56,57,58]. Basal layer KCs have lipid profiles rich in phospholipids (PLs) (70%), CH (13%), and triacylglycerides (TGs) (11%). In the granular layer, KCs produce glucosylceramides, PLs, and sphingomyelin (SM), which are stored in lamellar bodies and later metabolized to form the final lipids in the SC, including FFAs, CH, and CERs [46]. The FFAs, primarily saturated, contribute to the acidity of the SC [59]. These keratinocyte-derived lipids combine with sebum lipids, which include TGs, wax esters, squalene, and FFAs from sebaceous glands [60,61]. Sebum is crucial for waterproofing the skin, enhancing the barrier function, and supporting cutaneous microbiota regulation [61,62,63,64].

## 3. Lipids in the Skin

Cutaneous lipids are not only structural components of the skin’s physical barrier; they also function as active signaling molecules within a complex network involving epidermal and dermal constituents [65,66,67]. In response to environmental or signaling stimuli, membrane lipids generate bioactive mediators such as phospholipid metabolites, eicosanoids, endocannabinoids (ECBs) and CERs. These lipid messengers regulate physiological processes such as cell proliferation, differentiation, migration, melanogenesis and, when deregulated, are also involved in inflammatory skin diseases and cancer [68,69,70,71,72,73,74].

Since several reports have documented that lipid alterations and chronic inflammatory dermatoses, such as atopic dermatitis, psoriasis or acne, are related [75,76,77,78,79,80,81], lipidomics alone or in combination with metabolomics may provide valuable insights into the disease mechanism. Indeed, cutaneous lipid metabolism results in a continuous flux of bioactive species, and the lipid balance within the skin is finely tuned by metabolic perturbations or activation of the innate and adaptive immune systems.

For these reasons, skin lipid sampling and analysis are challenging tasks and the translational role of cutaneous lipidomics is becoming increasingly relevant, driving the development of lipidomic technologies. Recently, the sampling, extraction and analysis procedures of surface lipids have been investigated and standardized to harmonize the results of lipidome analysis in inflammatory skin diseases [82]. Although lipid signaling is associated with melanin production, the study of the role of cutaneous lipids in the process of melanogenesis under physiological conditions or in association with pigmentary disorders is a topic that is still partially explored and requires further investigation.

## 4. Lipid Metabolism and Analysis in Skin Pigmentation

As the largest organ in the body, the skin is exposed to a wide range of external signals, including UV radiation, biological and chemical factors (e.g., microorganisms, allergens and pollutants), and mechanical trauma, all of which affect skin physiology. UV radiation is one of the main environmental stressors that lead to detrimental effects such as skin cancer and aging. Notably, in response to these stimuli, the skin activates a complex neuro-immuno-endocrine system through the local release of various mediators (hormones, neuropeptides, cytokines/chemokines, serotonin, melatonin, cannabinoids, and steroids). This makes the skin a key player in linking environmental stimuli with the central nervous system and other organs, allowing local and global homeostasis [83,84]. 

Pigmentation is one of the skin’s main functions and is a highly intricate process influenced by genetic and environmental factors [85,86]. Melanogenesis is the result of cooperation of different cutaneous cell populations, including melanocytes (MCs), KCs, fibroblasts (HFs) and immune cells working in a coordinated manner [87]. Deregulations in one or more of these components and/or interactions can lead to homeostatic imbalance, resulting in pigmentary disorders. The key challenge is to understand how these molecular interactions unfold over time and contribute to the melanogenesis process.

### 4.1. Intrinsic/Extrinsic Factors in Skin Pigmentation

Melanocytes possess biosynthetic machinery to produce melanin within melanosomes [88]. Melanin synthesis involves the successful coordination of metabolic pathways across multiple intracellular compartments including the melanosome, mitochondria, ER/Golgi, and cytoplasm. While pigment production offers a communal protection from UV damage, the process also requires anabolic and redox demands that must be carefully managed.

The mechanism by which tyrosine is taken up into melanosomes is not fully understood. α-Melanocyte Stimulating Hormone (α-MSH) stimulates the melanogenesis pathway, increasing tyrosine uptake into melanosomes [89]. SLC7A5, a MITF target and L-type amino acid transporter (LAT1), has been suggested as a possible transporter for tyrosine. Inhibition of SLC7A5 causes pigmentation loss in certain cells, but its exact localization in melanosomes has not been confirmed [90]. Further studies are needed to clarify how tyrosine is transported into melanosomes.

Tyrosinase (TYR; EC 1.14. 18.1) is the rate limiting enzyme that catalyzes the initial steps of melanin synthesis using tyrosine as a substrate in melanosomes [91,92,93]. Tyrosine can be obtained from the diet or produced from phenylalanine through the enzyme phenylalanine hydroxylase (PAH; EC 1.14.16.1). This enzyme is also present in melanocytes, suggesting that they can produce tyrosine independently [94]. PAH is activated also in keratinocytes in response to UV irradiation, thereby increasing the tyrosine supply for melanogenesis [95]. The production of TYR is regulated by environmental stimuli, signaling pathways, and transcription factors, but it requires proper post-translational modifications and cofactors for successful synthesis. These modifications mainly occur in the ER and Golgi, and after processing, TYR is transported to the membrane of melanosomes, which are responsible for melanin synthesis, storage, and transport [96]. In melanosomes, TYR catalyzes the conversion of tyrosine to L-DOPA, which is further converted into L-Dopaquinone (DQ). DQ is a precursor that can form either eumelanin or pheomelanin. Eumelanin, which is typically brown or black, is produced when the ratio of DQ to cysteine availability is high. This leads to the spontaneous cyclization and oxidation of DQ to form DOPAchrome [97]. On the other hand, pheomelanin, which appears yellow to reddish-brown, is produced when cysteine is more available [98]. Cystine is imported into melanocytes through the xCT SLC7A11 transporter [99], where it is reduced to cysteine by cystine reductase. The cysteine is then transported into melanosomes by the MFDS12 transporter. Inside the melanosome, cysteine is incorporated into DQ to form cysteinyl dopa isomers, which are further oxidized to cysteinyl-DQ [100]. This cysteinyl-DQ undergoes cyclization and rearranges into benzothiazine and benzothiazole intermediates, which then polymerize to form pheomelanin [101].

In addition to being precursors and intermediates in the melanogenesis process, it is well known that tyrosine and DOPA act as skin homeostasis regulators at the cellular, tissue, and systemic levels. This extends the function of melanin-producing cells to neuro-immuno-endocrine effectors [102]. Moreover, melanin and melanogenesis may be viewed as a double-edged sword. In addition to having a protective role, they can shift towards a detrimental effect favoring tumor progression and therapy resistance when deregulated [103]. 

In addition to the role of UV radiation as an extrinsic factor, skin pigmentation is also stimulated by several intrinsic factors derived from melanocytes, keratinocytes, dermal fibroblasts, and endothelial cells. These factors trigger the process of melanogenesis, the transport of melanosomes within melanocytes and their transfer to neighboring keratinocytes.

Skin pigmentation requires close intercellular communication, and exosomes, endosome-derived extracellular vesicles, facilitate the direct transfer of proteins, lipids, and RNAs between epidermal cells [104,105,106]. Exosomes have a role in keratinocytes/melanocytes crosstalk in the epidermal melanin unit [107]. Several studies have shown that keratinocyte-derived exosomes not only promote melanocyte proliferation and dendricity but also increase tyrosinase activity and melanin production [108,109,110].

Hormonal factors regulate melanogenesis acting in different ways depending on the species, the cellular compartment, and the physiological or pathological context [111,112]. The biology of proopiomelanocortin (POMC) and the peptides derived from it through proteolytic digestion, as well as the hypothalamus–pituitary axis, play a crucial role in UVR-induced pigmentation. Melanocortins are a family of neuroendocrine peptides originally described as regulators of cutaneous pigmentation and cortisol production. This family includes melanocyte-stimulating hormone (MSH) peptides (α-, β- and γ-MSH), adrenocorticotropic hormone (ACTH), β-lipoprotein and β-endorphin peptides. By binding to G protein-coupled receptors (GPCRs), such as melanocortin receptors (MC1-MC5) and opioid receptors, these neuropeptides play a key role in melanogenesis, cytoprotection, skin barrier function, and the immune system [83]. α-MSH is released from keratinocytes and binds to the melanocortin-1 receptor (MC1R). MC1R, a G-protein-coupled receptor, is expressed on the membrane of melanocytes. The interaction between this hormone and receptor increases cAMP levels through the activation of adenylate cyclase, which triggers a downstream pathway involving the activation of protein kinase A (PKA; EC 2.7.11.11), CREB, and MITF transcription factors. MITF is a key regulator of the expression of numerous pigmentation-associated genes and melanin synthesis enzymes [96,113,114,115], promoting melanogenesis [116,117,118,119]. The MC1R gene also causes differences in skin and hair color by controlling the ratio of pheomelanin to eumelanin synthesis [120,121,122,123,124,125]. Other keratinocyte-derived soluble factors that induce melanogenesis via paracrine action include endothelin-1 (ET-1), basic fibroblast growth factor (bFGF), stem cell factor (SCF), hepatocyte growth factor (HGF), granulocyte-macrophage colony-stimulating factor (GM-CSF), nerve growth factor (NGF), and prostaglandins (PGE_2_/PGF_2α_) [126].

Fibroblasts produce pro-melanogenic factors such as HGF, SCF, and bFGF, but also release inhibitory factors like Dickkopf 1 (DKK1) and transforming growth factor-beta 1 (TGF-β1). Endothelial cells release α-MSH, ET-1, and prostaglandins (PGs) [127]. Mast cells secrete α-MSH, GM-CSF, nitric oxide (NO), and histamine. Inflammatory cells produce both pro-melanogenic factors, like PGs, leukotrienes (LTs), and thromboxanes (TXs), as well as cytokines (such as IL-1, IL-6, and TNF-α) that can suppress pigmentation.

### 4.2. Fatty Acids and Phospholipids

Intracellular FAs are required for the biosynthesis of biological membrane lipids, signaling molecules and post-translational modifications of proteins, and are also necessary to provide energy for cell survival and proliferation through the β-oxidation process [128]. Numerous studies have investigated the effects of different fatty acids on melanin production, often with conflicting results. These lipids, classified according to their chain length and degree of unsaturation, have different effects on melanogenesis, such as their ability to target the activity of TYR or transcription factors, including MITF. In vitro studies have generally shown that unsaturated fatty acids, including oleic acid (OA, C18:1), linoleic acid (LA, C18:2), alpha-linolenic acid (ALA, C18:3), arachidonic acid (AA, C20:4), and docosahexaenoic acid (DHA, C22:6), with medium to long chains of 18–22 carbons, inhibit melanin synthesis. These lipids regulate the degradation of TYR at the post-Golgi stage by modulating its ubiquitination and subsequent degradation via the ubiquitin-proteasome pathway. They may also interfere with melanogenesis-related signaling pathways, thereby contributing to their anti-melanogenic effects [129,130,131].

Docosatrienoic acid (DTA), also known as 22:3(n-3), inhibits nuclear translocation of MITF, reducing the transcription of melanogenic enzymes and melanin production. This suggests DTA as a potential treatment for skin pigmentation disorders [132]. In contrast, saturated fatty acids, such as palmitic acid, with a short-chain structure of 16 carbons, enhance melanin production by stabilizing TYR and promoting melanosome maturation [130].

Metabolic changes associated with pigmentation were investigated using a B16 cell-autonomous pigmentation model [133]. Integrated transcriptomic and metabolomic analyses identified three synchronized phases of melanocyte pigmentation: preparatory, melanogenic, and recovery. The melanogenic phase was associated with heightened fatty acid biosynthesis, lipid droplet accumulation and high levels of glycolysis. This increase was attributed to the activation of Sterol Regulatory Element Binding Transcription Factor 1 (SREBF1), a key lipid mediator that is stimulated by several pigmentation activators, including α-MSH.

Activation of SREBF1 increases fatty acid synthesis, resulting in the formation of triacylglycerols (TAGs). Lipid droplets in melanocytes are rapidly utilized by β-oxidation during the melanogenic phase. Previous studies have shown an accumulation of these droplets in melanocytes derived from UV-exposed skin areas [134]. Analysis of mitochondrial respiration showed that during pigmentation melanocytes have a higher spare respiratory capacity for fatty acid utilization, allowing them to use this pathway during periods of increased energy demand. However, the reliance on fatty acids may lead to defective mitochondria during the recovery phase. This is consistent with previous reports showing decreased mitochondrial respiration in hyperpigmented cells [135]. The shift to glycolysis and the depletion of lipid droplets suggest that these cells become dependent on anaerobic glycolysis to meet their energy needs during recovery phase. Lipid metabolism in melanocytes is regulated by peroxisome proliferator-activated receptors (PPARs) and SREBFs. Srebf1 knockdown results in a depigmenting effect in both B16 and primary melanocytes. Inhibitors targeting fatty acid synthase (FASN; EC 2.3.1.85), Diacylglycerol O-Acyltransferase 1 (DGAT1; EC 2.3. 1.20) and lipase also affect pigmentation, highlighting the importance of de novo fatty acid synthesis, storage, and lipolysis in melanogenesis. Acute UVA irradiation induces changes in the phospholipid profile of melanocytes, leading to a significant increase in the relative content of phosphatidylcholine (PC), phosphatidyloethanolamine (PE), phosphatidylinositol (PI), and sphyngomyelins (SM) species, suggesting their involvement in the melanogenic process [136,137]. The induction of PLC-PPAR-γ axis in connection to α-MSH stimulation (with the generation of fatty acids, such as linoleic acid or arachidonic acid) strongly suggests a link between MC1R activation and lipid mediators. In turn, linoleic acid and arachidonic acid trigger cascade reactions mainly related to the control of proliferation and inflammation [138,139,140], interfering also with pigmentation. This evidence highlights the pleiotropic action of activated MC1R, not uniquely related to melanogenic effects [141,142]. Lipidomic analysis showed that in primary melanocytes chronically exposed to UV radiation at a UVA/UVB ratio similar to that in solar light, lipid raft components such as SMs, as well as other signaling molecules such as PIs, progressively decreased over time [136]. In contrast, TGs associated with energy storage progressively increased, which could be interpreted as a survival mechanism in adverse conditions. These changes suggested strong effects on important biological functions.

### 4.3. Cholesterol

The role of cholesterol (CH) homeostasis in regulating melanogenesis is not yet fully understood. Some evidence indicates that CH plays a pro-melanogenesis role in human epidermal melanocytes, where it is found in the plasma membrane. Melanocytes can synthesize and uptake cholesterol via the LDL receptor/Apo-100 pathway, in an autocrine manner. Cholesterol (CH) binds to estrogen β receptors, stimulating cAMP release and activating the CREB/MITF/tyrosine hydroxylase/tyrosinase pathways involved in pigmentation [143]. Additionally, it is believed that cholesterol protects the key enzyme tyrosinase from proteasomal degradation by stabilizing the cell membrane. Therefore, it was expected that lowering cholesterol would potentially inhibit melanogenesis. Hydroxymethylglutaryl-CoA reductase (HMG-CoA reductase; EC 1.1.1.88) is a key enzyme in the cholesterol biosynthetic pathway, and molecules capable of inducing the downregulation of C/EBP (CCAAT/enhancer-binding protein) and the subsequent reduction in HMG-CoA reductase expression have been found to inhibit melanogenesis [144]. Furthermore, oxysterols—oxygenated derivatives of cholesterol that play a pivotal role in cholesterol homeostasis—have been shown to decrease pigmentation in murine melanocytes. A significant pigmentation reduction requires an oxygen-bearing functional group in the C20 or C25 position, with 25-hydroxycholesterol (25HC) presenting the strongest effect. The oxysterol mechanism of action on pigmentation does not occur via liver X receptor (LXR) activation, but rather through the enhancement of tyrosinase degradation post-translationally in the Golgi compartment [145]. Moreover, recent evidence suggests that 7-dehydrocholesterol (7-DHC), the precursor of cholesterol and the starting point for vitamin D3 formation in the skin after UVB-induced photo-transformation, plays a role in regulating melanocyte activities. Photoderivatives of 7-DHC, such as lumisterol 3 (L3) and tachysterol 3 (T3), can be enzymatically converted in biologically active hydroxy metabolites able to affect various melanocyte functions. However, their role in regulating melanogenesis remains unclear [146]. 

### 4.4. Bioactive Lipids

#### 4.4.1. Eicosanoids

The eicosanoid PGs and LTs are lipid signaling factors derived from the metabolism of arachidonic acid via the cyclooxygenase (*COX*; EC 1.14.99.1) and lipoxygenase pathways, respectively. PGs are key mediators of diverse functions in the skin and several reports suggest that PGs mediate post-inflammatory pigmentary changes through modulation of melanocyte dendricity and melanin synthesis [147]. Although PGE_2_ is mainly secreted by keratinocytes, melanocytes also have the machinery for its production through the activation of the major isoforms of COXs: COX-1, which is constitutively expressed to maintain physiological levels of PGs, and COX-2, which is induced by various inflammatory stimuli such as cytokines, growth factors and UV radiation [148]. In addition, the expression of COX-2 seems to be upregulated by α-MSH, thereby increasing the production of PGs [148]. PGE_2_ acts in a paracrine and autocrine manner to regulate melanocyte proliferation and function by increasing cAMP levels, activating MITF and consequently the expression of tyrosinase, tyrosinase-related protein 1 (TYRP1; EC 1.14.18-) and tyrosinase-related protein 2 (TYRP2/DCT; EC 5.3.3.12) [148,149]. PGE_2_ binds to four receptors (EP1–4) belonging to the G-protein-coupled membrane receptors [148,150], the activation of which has opposite effects. Indeed, the activation of EP2 and EP4 stimulates melanogenesis, whereas EP3 inhibits pigmentation by decreasing cAMP levels [147,148]. It has been reported that epidermal melanocytes from subjects with different phototypes respond to UVB or arachidonic acid stimulation by upregulating PGE_2_ production, reflecting the cells’ intrinsic membrane biochemistry and/or the activity of the PGE_2_-producing machinery [151]. However, it has been proposed that UVB-induced PGE_2_ production is not related to intrinsic melanogenesis capacity and suggests that skin phototype appears to be determined by the nature of the keratinocyte partner in the epidermal-melanin unit [151]. In addition, melanocytes express the PGF_2α_ receptor FP, a G-protein coupled receptor that activates multiple signaling pathways, including phospholipase C-induced phosphoinositide turnover, intracellular calcium mobilization, mitogen-activated protein kinase activation, and protein kinase C activation. Studies have shown that UV radiation regulates the FP receptor in melanocytes both in vitro and in human skin in vivo. UV irradiation stimulates melanocytes to produce PGF_2α_, which binds to the FP receptor and activates signaling pathways that promote dendricity and pigmentation in melanocytes, suggesting that it acts as an autocrine factor to regulate melanocyte differentiation [147,149]. Other arachidonate-derived lipid mediators, such as LTs, including LTC4 and LTD4, and TXs, including TXB2, are also able to increase dendricity and tyrosinase levels in melanocytes and have been suggested as key players in post-inflammatory skin pigmentation [152].

#### 4.4.2. Sphingolipids

Sphingomyelinase (EC 3.1.4.12), found in cell membranes, plays a role in cell signaling by metabolizing the phosphodiester bond in SM, and forming phosphocholine and CERs. The latter are then either reconverted to SMs by SM synthase [153] or are broken down into sphingosine and fatty acids by ceramidase [154]. UV irradiation accelerates the formation of CERs in skin cells through the hydrolysis of sphingomyelin and de novo synthesis [155,156,157]. CERs inhibit melanogenesis through multiple pathways. They reduce DNA synthesis in the S phase in a dose-dependent manner and affect the activation of ERK and Akt/PKB pathways, leading to a significant reduction in melanin content in Mel-Ab cells [158]. Additionally, ceramides decrease MITF gene and protein expression and cause delayed activation of ERK and later activation of Akt/PKB, resulting in a sustained suppression of melanogenesis [159,160]. Spingosine-1-phosphate (S1P), a metabolic derivative of CER, produced by sphingosine kinase [161], is secreted by cells, acts as a signaling molecule in the organism, and has also been studied for its anti-melanogenic properties [162,163]. It directly inhibits intracellular tyrosinase activity in a dose-dependent pattern and reduces MITF expression via activation of the ERK signaling through the S1P receptor3 pathway [159,164].

#### 4.4.3. Endocannabinoids

ECBs are endogenous fatty acid amides and monoacylglycerols, of which anandamide (AEA) and 2-arachidonoylglycerol (2-AG) are the best known [165]. These molecules bind to cannabinoid receptors CB1 and CB2, and AEA also binds to TRPV1 channels [166,167]. The biological activity of the eCBs is regulated by metabolic processes, including Ca^2+^-dependent biosynthesis catalyzed by NAPE-PLD for AEA and by a specific phospholipase C for 2-AG, and their degradation by fatty acid amide hydrolase (FAAH; EC 3.5.1.99) for AEA and monoacylglycerol lipase (MAGL; EC 3.1.1.23) for 2-AG [168,169,170,171]. Human keratinocytes and melanocytes have a fully functional endocannabinoid system (ECS), including AEA and 2-AG, their target receptors (CB1, CB2, and TRPV1), and their metabolic enzymes [172]. Non-cytotoxic doses of eCBs have been shown to enhance melanin synthesis through CB1 receptor-dependent activation of tyrosinase gene expression [173]. This process is mediated by p38 and p42/44 MAPKs, CREB, and the regulator MITF, without affecting cAMP levels. Since AEA or 2-AG can be rapidly released from cell membranes through enzymatic hydrolysis of phospholipid precursors, eCB-induced melanogenesis may offer a faster alternative to the α-MSH-dependent pathway for melanin production. Considering that keratinocytes surround melanocytes in a ratio of ~35:1, their ability to synthesize eCBs makes them a source of AEAs for the rapid activation of melanogenesis. In response to a typical pro-melanogenic stimulus, such as UVB exposure, the levels of AEA and 2AG are increased. This suggests that keratinocytes may release CB1-binding eCBs to induce melanin synthesis in nearby melanocytes as a protective response for the keratinocytes themselves.

#### 4.4.4. Lipidomics for Studying Melanogenesis

Given the key role of lipid signaling in the regulation of both basal and externally induced melanogenesis, analysis of the lipid profile using mass spectrometry techniques is a valid tool both for detailed study of the involvement of lipids in melanogenesis and for facilitating accurate diagnosis of skin lesions characterized by pigmentary changes (Table 1). Recently, mass spectrometry imaging-based lipidomic approaches have been explored to address the challenging diagnosis of cutaneous melanocytic lesions, ranging from benign melanocytic nevi to malignant melanoma. Matrix-assisted laser desorption/ionization imaging mass spectrometry (MALDI-IMS) was used to monitor disease-specific lipid changes in situ, linking histology to the spatial distribution of molecules in tissues [174]. Along a section of a nevus biopsy, each lipid species shows a characteristic distribution in the epidermis or dermis, with a notable abundance of low unsaturated SM species, especially in the dermis, highly unsaturated PE and PE plasmalogens (PE ether [alkyl acyl]/PE ether [alkenyl acyl]) in melanocytes, and mono- and di-unsaturated molecular species of PI in the epidermis. Specific lipid profiles correspond to melanocytes with different degrees of maturation. Since melanocytes “mature” with depth in benign but not in malignant lesions, studying the metabolic profile of melanocyte clusters may be a useful approach to diagnosis. In addition, desorption electrospray ionization (DESI) coupled with compact post-photoionisation assembly (DESI/PI) has been developed for comprehensive polar/non-polar lipid imaging to delineate melanocytic nevi within normal tissue and to find potential lipid biomarkers [175]. Using DESI and DESI/PI mass spectrometry methods, various lipids were visualized and some distinct lipids could distinguish melanocytic nevi from normal tissue, such as cholesterol, S1P, PCs, PEs, FFAs, MAGs, and DAGs. These lipids were found to be statistically significant in melanocytic nevi and normal tissue, with cholesterol being the most significant biomarker in melanocytic nevi.

## 5. Lipid Metabolism and Analysis in Hyperpigmentary Disorders

Melanin synthesis is essential for sun protection and skin cancer prevention, but excessive production can lead to hyperpigmentation disorders [128,176,177,178,179,180]. These conditions are common across different ethnicities, including Caucasians, Africans, and Asians [85,181,182]. Various factors can trigger hyperpigmentation, including the use of certain drugs (e.g., antibiotics or contraceptives) and hormonal changes (e.g., estrogen or progesterone), which stimulate melanin production in response to sun exposure. Inflammatory conditions, such as those found in acne lesions, can also trigger hyperpigmentation.

Post-inflammatory hyperpigmentation (PIH) can be caused by inflammatory skin diseases including acne vulgaris, atopic dermatitis, psoriasis and lichen planus, as well as injuries such as burns and chemical exposure. PIH is an acquired hypermelanosis that can affect anyone, regardless of age or gender. All skin phototypes can be affected, although it is most prevalent among individuals with dark skin (Fitzpatrick skin types IV–VI). PIH is characterized by dark macules or patches that typically appear in the same area as the initial skin injury, resulting from increased melanin synthesis and transfer to keratinocytes [183,184,185]. Hyperpigmentation is closely linked to the production of arachidonic acid metabolites, including prostaglandins (PGE2 and PGD2), leukotrienes (B4, LTC4, LTD4 and LTE4), thromboxane B2 (TXB2) and 12-hydroxy eicosatetraenoic acid (12-HETE). These metabolites, along with cytokines (IL-1, IL-6 and TNFα), reactive oxygen and nitrogen species released during the inflammatory response, stimulate melanocyte proliferation and activity leading to abnormal melanin production [186].

Skin aging leads to hyperpigmentary disorders like solar lentigo (SL). This process is regulated by intrinsic genetic and hormonal factors. Decreased corticosteroids, sex steroids (estrogens), and their receptors are involved in skin changes over time [187,188,189]. External factors like biological and chemical agents, air pollution, smoking, stress, lifestyle, and mainly UV radiation affect skin aging.

The UV-induced inflammatory state is responsible for cell damage and photoaging through the production of reactive oxygen species (ROS) and the release of arachidonate-derived mediators [190,191]. Specifically, UV-associated oxidative stress activates cytoplasmic phospholipase A2 (cPLA2), the enzyme that hydrolyses AA from membrane phospholipids. Free AA is in turn converted by COXs into its metabolites, including prostaglandins (PGE2, PGF2α, PGD2), prostacyclin (PGI2) and TXs (TXA2, TXB4), and by lipooxygenases (LOXs; EC 1.13.11) into LTs (LTB4, LTC4, LTD4, LTE4) [192,193]. Induction of COX-2 in response to UV exposure leads to the production of PGE_2_, which exerts effects on pathophysiological processes in the skin, playing a role in tumor development, inflammation, immune function, wound healing and also pigmentation [147,148,149].

Based on the crucial role of COX-2 in stimulating pigmentation, some studies have investigated the possibility of acting on the COX-2/PGE_2_ axis to suppress melanogenesis [194,195,196,197]. Kim et al. showed that knocking down COX-2 in melanocytes can be effective in reducing the expression of tyrosinase, TRP-1/2, gp100 and MITF and in decreasing tyrosinase activity. COX-2 inhibition was also able to downregulate the synthesis of melanin induced by stimulation with αMSH [148]. COX-2 inhibitors may therefore be good candidates for the development of anti-melanogenic drugs for the successful treatment of hyperpigmentary disorders.

Since skin affected by atopic dermatitis [198] and psoriasis [199,200] shows upregulation of the inflammatory mediators PGE2 and LTB4, inhibition of the COX and lipoxygenase pathways may be effective in the prevention and/or treatment of post-inflammatory hyperpigmentation.

In addition, UV radiation exerts effects on the endocannabinoid system, increasing the expression of AEA and 2-AG receptors (CB1/2 and TVRP1) [201,202,203]. CB2 may play a critical role in inducing antioxidant and anti-inflammatory responses [204]. ECBs action also involves the activation of Nrf2, which regulates the expression of antioxidant enzymes that protect the skin from UV damage [205]. The use of exogenous antioxidants such as ascorbic acid and rutin, which interact with the endocannabinoid system, has been shown to protect against lipid peroxidation and reduce UV-induced inflammation [202].

SL and melasma are common in sun-exposed areas and appear as dark spots on the skin. While both are linked to excess melanin, SL is more common in elderly people and typically presents as light-to-dark brown marks on the face and hands.

Histologic analysis showed two different patterns: a flattened, thinner epidermis with basal melanosis and elastosis or epidermal hyperplasia with elongated rete ridges and pigmented basaloid cells [206,207]. Lesions on sun-exposed areas show strong links to long-term UV exposure [187,206,208]. Chronic UV exposure deregulates the pigmentary system by upregulating melanogenesis, promoting cellular senescence, and inducing inflammation [208].

Increased cellular senescence has been demonstrated in SL lesions. Keratinocytes expressing a senescence-associated secretory phenotype (SASP) show reduced proliferation, deregulated differentiation, and hyperplasia, which underlie the increased epidermal thickness and enhanced melanogenesis [187,209,210,211]. Moreover, keratinocyte senescence may impair autophagy, reducing melanosome degradation [212]. Senescent dermal fibroblasts (p16INK4A-positive) accumulate at pigmented lesion sites [213,214], contributing to SL.

Senescent fibroblasts promote hyperpigmentation by secreting pro-inflammatory cytokines (e.g., IL-1α, IL-1β, TNFα IL-6, IL-8), matrix metalloproteinases (*MMPs*; EC 3.4.24), pro-melanogenic factors (e.g., HGF, KGF, SCF), and other factors like stromal cell-derived factor (SDF)-1 and growth/differentiation factor (GDF)-15 [214]. Studies by Yoon et al. suggest that reduced expression of SDF-1 or CXCL12 in senescent fibroblasts may prevent inhibition of the cAMP/MITF pathway in melanocytes [213]. Additionally, CCL2 released from senescent fibroblasts stimulates monocytes to increase PGE2 levels, promoting melanogenesis. UV exposure increases dermal blood vessel size and number by enhancing VEGF expression, which prompts endothelial cells to release pro-pigmentation factors like ET-1/ENDRB and iNOS.

Several studies have analyzed gene expression profiles in skin biopsies from SL lesions. In a mouse model of pigmented lesions, increased expression of chemokine genes was induced by high levels of interferon-γ (IFN-γ), which promotes the migration of inflammatory cells and activates melanocyte functions [215]. DNA microarray analysis of SL lesional skin showed upregulation of genes related to inflammation and fatty acid metabolism, while genes associated with terminal differentiation and keratinized envelope were downregulated [210]. Jeong et al. conducted a multi-system level analysis using RNA-seq data from inflammatory and non-inflammatory SL skin samples [208]. They identified upregulation of genes involved in oxidative stress (e.g., NF-kB), extracellular immunity, mitochondrial innate immunity, and CXCR3 in inflammatory SL, suggesting CXCR3′s role in melanin synthesis and the activation of the JAK-STAT pathway. In non-inflammatory SL, they found upregulation of genes that protect against UV damage, such as SLC6A9, which encodes a glycine transporter involved in UVB-induced senescence protection [216].

Melasma, the other acquired pigmentary disorder, mainly affecting women of childbearing age, is characterized by asymptomatic light to dark brown spots with irregular edges in the photo-exposed areas, especially the face [85,217,218,219,220]. It is a complex disorder with markers of photoaging [188,219,220,221], and its lesions show a high degree of UV and visible light-induced damage, such as solar elastosis, basement membrane modifications, chronic inflammation and increased vascularity [218,222,223,224,225,226,227]. Keratinocyte- and fibroblast-derived paracrine melanogenic factors are over-expressed, suggesting an impairment of the intercellular network at the basis of deregulated skin pigmentation [224,228,229,230,231].

Several data in the literature support the involvement of lipids in the pathogenesis of melasma, both with a structural function, such as epidermal lipids involved in the formation of the skin barrier, and as bioactive mediators. Kang and co-authors [232] performed a transcriptomic study to identify genes differentially expressed in melasma lesional and perilesional skin sites. Most of the lipid metabolism-related genes were downregulated in lesional areas, suggesting a relationship with the observed impairment of barrier function. Bioinformatic analysis in melasma lesional skin identified a significant upregulation of genes involved in prostaglandin synthesis. Furthermore, genetic and immunohistochemical analyses revealed significant induction in the expression of COX-2 in melasma-affected skin compared to healthy skin.

In addition, COX-2 expression positively correlates with solar elastosis, epidermal melanin and the Melasma Activity and Severity Index score [233]. Lee and co-authors [212] confirmed that melasma skin is characterized by impaired stratum corneum integrity and delayed barrier recovery. The authors also showed a trend towards reduced protein expression of PPARα, an important regulator of lipid catabolism, in lesional skin. Chung and co-authors [234] also described the downregulation of lipid genes related to the PPAR signaling pathway and the upregulation of genes involved in stratum corneum barrier function in melasma lesions, revealing significant differences from normal skin. Recently, tape stripping and compositional analysis of skin barrier-related epidermal lipids revealed some abnormalities in female melasma [235]. Compared with non-lesional skin, the epidermal thickness and the content of total lipids, phosphatidic acid, phosphatidylserine and CERs were significantly increased in the lesional skin of melasma patients. In particular, the expression of very long chain (VLC) and ultra-long chain (ULC) CERs, which are important components of the lipid barrier [236], was significantly increased in lesional skin, possibly as a compensatory mechanism to maintain skin barrier function. These data are further supported by the study by Zhu and co-authors [237] who analyzed the lipidome profiles of skin surface lipids in patients with melasma before and after treatment with oral tranexamic acid and topical hydroquinone cream to investigate possible alterations in lipid composition. The treatment not only effectively reduced melanin content and blood vessels in melasma lesions, but also reduced total lipid, phosphatidylcholine, and phosphatidylethanolamine levels, suggesting a role for lipids in the development of the disease. Sultan and co-authors [133] showed that inhibitors targeting key lipid mediators such as SREBF1, FASN, DGAT1, and lipases can reverse hyperpigmentation in a guinea pig UV tanning model, confirming that de novo fatty acid synthesis, storage and lipolysis are integral to the execution of melanogenesis.

Melasma is more common on specific areas of the face, such as the malar area, forehead, and upper lips, which are rich in sebaceous glands (SGs). SGs not only synthesize and produce sebum but also secrete several mediators and participate in the multicellular communication network that regulates skin cell function [238,239,240,241]. Some data in the literature indicate that sebocyte-rich regions of the body, such as the face, axillae, and genitalia, have the highest melanocyte density and pigmentation [242]. Furthermore, co-culture of the human sebaceous cell line SZ95 and melanocytes promotes melanocyte proliferation and dendricity [243]. In addition, UV-oxidized skin surface lipids, particularly squalene derived from SG activity, induce melanocyte proliferation and melanin synthesis in organ tissue cultures [244]. Foolad and co-authors [245] observed a reduced rate of facial sebum excretion in melasma patients, suggesting a role for sebaceous regulation in the development of the disease. Recently, the involvement of SGs in the physiopathology of melasma has been demonstrated [246]. In particular, UVA-irradiated sebocytes increased the expression of PPARγ, a ligand-activated transcription factor with a key role in sebocyte differentiation and lipid metabolism [247] and exerted a significant paracrine effect by releasing lipid mediators such as arachidonic acid, LTB4, and prostaglandins (in particular PGD2, PGE2 and PGF-2α). These lipids are involved in inflammation, pigmentation and aging as SASP factors [248,249], capable of driving changes in fibroblast and melanocyte behavior [248,250,251], thus reinforcing the concept that increased melanocyte activity in melasma is linked to a senescence process also in the dermal compartment.

**Figure 1:**
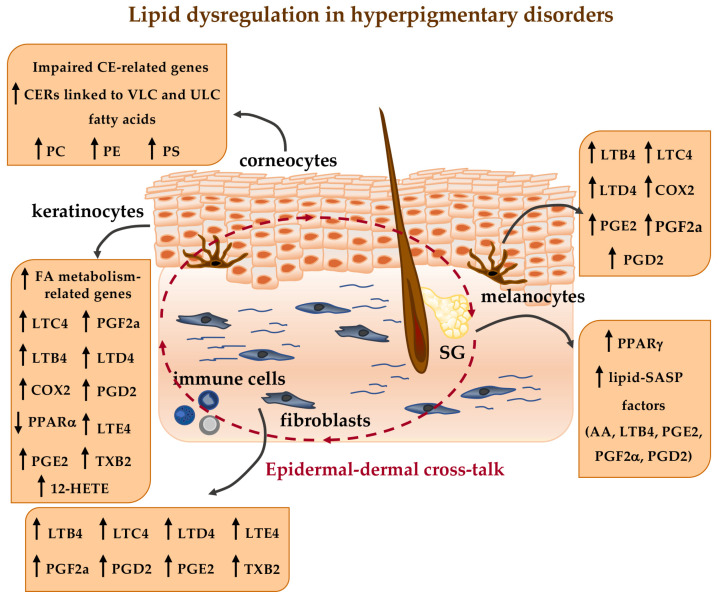
Schematic overview of lipid-related dysregulation occurring in hyperpigmentary disorders. CE: cornified envelope; CERs: ceramides; VLC: very long chain (VLC) Cers; ULC: ultra-long chain Cers; PC: phosphatidylcholine; PE: phosphatidylethanolamine; PS: phosphatidylserine; FA: fatty acid; LTB4: leukotriene B4; LTC4: leukotriene C4; LTD4: leukotriene D4; LTE4: leukotriene E4; TXB2: thromboxane B2; 12-HETE: 12-hydroxy eicosatetraenoic acid; COX2: cyclooxygenase-2; PGE2: prostaglandin E2; PGF2a: prostaglandin F2a; PGD2: prostaglandin D2; PPAR*α*: peroxisome proliferator-activated receptor *α*; PPARγ: peroxisome proliferator-activated receptor γ; SASP: senescence-associated secretory phenotype; AA: arachidonic acid; SG: sebaceous gland. The arrows in the Figure indicate the following: **↑** increased level of the indicated parameters; **↓** decreased level of the indicated parameters.

The preponderance of melasma during the reproductive lifespan of women, particularly in association with pregnancy, oral contraceptives, and hormone replacement therapy, indicates that female sex hormones, especially estrogens, are risk factors for the development and exacerbation of the disorder [217,221,252,253]. The literature data indicate that estrogens stimulate melanocyte and keratinocyte pro-pigmentary factors when combined with other stimuli, such as UVR, thereby supporting the photoaged environment associated with melasma [217,246,254]. To date, no studies have directly analyzed the influence of female sex hormones on lipid alterations in melasma. However, recent findings examining the relationship between hormone replacement therapy and menopausal changes in the epidermis indicate that estrogens play a role in keratinocyte ceramide metabolism and epidermal barrier regulation [255]. These data suggest that hormonal fluctuations during pregnancy and oral contraceptive use can influence ceramide abundance and profile, potentially contributing to the alterations in this class of lipids observed in melasma lesions (Figure 1).

## 6. Lipid Metabolism and Analysis in Hypopigmentary Disorders

Vitiligo, the most common hypopigmentary skin disorder, is an acquired disease characterized by a progressive loss of melanocytes. The pathogenesis of the disease is puzzling and not yet fully explained. It is clear that multiple mechanisms coexist and interact, leading to the activation of autoimmune responses that culminate with the CD8+ cell-mediated death of melanocytes [256,257]. Although the major macroscopic manifestation of vitiligo is the appearance of white patches, it is now recognized that alterations also occur in normal-appearing skin and at a systemic level [258,259].

In this challenging etiopathogenetic scenario, early studies had already revealed the involvement of lipid alterations in vitiligo. Notably, vitiligo melanocytes have been shown to have defective content of the mitochondrial inner membrane phospholipid cardiolipin. Such intrinsic lipid impairment, combined with increased production of ROS and reduced activity of mitochondrial electron transport chain proteins, has been suggested to be the basis of the progressive degeneration of melanocytes [260]. The same membrane cardiolipin defects associated with increased cholesterol levels have also been detected in peripheral blood mononuclear cells, suggesting that structural and functional changes in membrane lipids may represent markers of the disease [261]. Defective mitochondrial respiratory responses and higher ROS production in combination with changes in lipid profiles have also been observed in the PIG3V immortalized cell line derived from peri-lesional vitiligo skin, further pointing at altered cellular metabolism in vitiligo [262]. Increased amounts of CH and its oxidative derivatives 7-beta-hydroxycholesterol and 7-ketocholesterol has also been observed in non-lesional vitiligo fibroblasts, demonstrating that an altered lipid composition is a common feature of several cell populations in patients with vitiligo [263].

Evidence linking autoimmunity, oxidative stress and lipid metabolism in vitiligo is now continuously growing thanks to ongoing advances in the omics sciences of metabolomics and lipidomics. Specifically, the lipidomic perspective has allowed the identification of a broad panel of lipids that are dysregulated in vitiligo. As a result, this approach has become a valuable resource not only for investigating the pathogenetic mechanisms of the disease but also for identifying markers of its occurrence and activity.

Liang et al., by performing lipidome profiling combined with metabolomic and proteomic analyses of plasma samples from vitiligo patients and healthy controls, identified 22 lipids that are aberrantly expressed in vitiligo. Among these, the inflammatory mediators lysophosphatidylcholine and PAF were found to be upregulated, suggesting their role as biomarkers of disease progression [264].

A comprehensive lipidomic analysis of the stratum corneum performed by our group revealed a prominent decrease in CER levels and an enrichment in FFA, CH, and CHS in vitiligo patients in comparison to controls, highlighting an improper composition of the skin barrier lipids. These alterations parallel the defective expression of several enzymes regulating skin barrier lipid metabolism such as CERS3 and ELOVL4 observed in primary cultures of non-lesional keratinocytes induced to differentiate in vitro [265]. These findings suggest a role for ceramides as messenger molecules that regulate cellular processes such as cell cycle arrest, differentiation, and apoptosis.

Dysregulated fatty acid metabolism is a defining metabolic alteration involved in the pathogenesis of vitiligo [266]. Circulating FFAs such as ALA, AA, and eicosapentaenoic acid (EPA) are related to several autoimmune diseases. However, the exact category of fatty acids involved in vitiligo and their functional effect on CD8+ T cells are still undefined. Ye and co-workers demonstrated that serological levels of ALA were significantly upregulated in vitiligo patients and that their concentrations may partly reflect the severity of the disease (VASI scores) [267]. On the contrary, ARA levels tended to decrease in vitiligo. However, supplementation with ARA could inhibit the activation of CD8+ T cells and the secretion of INF-γ granzyme B and perforin [267], similar to the contribution of EPA supplementation in the recovery of autoimmune diseases such as rheumatoid arthritis and systemic lupus erythematosus [268,269].

By integrating genomic and metabolomic analyses, a recent study revealed that metabolites associated with vitiligo are mainly enriched in alpha-linolenic and linoleic acid (LA) metabolism and established a causal relationship between metabolites and disease risk [270]. ALA was assessed as a risk factor and AA as a protective element. In particular, AA inhibited the proliferation, activation, and function of CD8+ T cells in vitro. The potential link between vitiligo and fatty acids has been further confirmed by their role in other immune diseases. Low plasma levels of LA and ALA [271] could reduce the secretion of pro-inflammatory cytokines by T cells and keratinocytes in psoriasis [272].

Changes in circulating FA composition associated with deregulated activity of their biosynthetic enzymes have also been recently described by our group in a cohort of 50 patients with vitiligo. Specifically, we observed an increase in pro-inflammatory n6 series FAs and a decrease in anti-inflammatory n3 series FAs, indicating the presence of low-level persistent inflammation and further supporting the systemic nature of vitiligo [273]. In addition, parameters related to altered lipid metabolism including high serum total cholesterol, elevated levels of triglycerides and low-density lipoprotein (LDL) and lower high-density lipoprotein (HDL) have now been frequently reported in patients with vitiligo [274,275]. In this regard, accumulating evidence points to an association between vitiligo, primarily the active/severe form, and metabolic syndrome [276,277,278], a condition in which an impairment of lipid metabolism constitutes a characterizing trait [279].

The high translational feasibility of lipids in therapeutic strategies is recently well reported by An and co-workers whose study clearly demonstrated that in mouse models PGs limited T-cell infiltration in UVB-irradiated skin tissue in combination with the immunosuppressant drug ruxolitinib [280]. Several studies have shown that topical application of prostaglandin analogs such as latanoprost and bimatoprost, either used alone or as add-on NB-UVB phototherapy and microneedling to enhance the absorption of topical agents, can be effective alternatives therapies for vitiligo [281,282,283]. Previously, Anbar et al. demonstrated that topical latanoprost alone produced a clinical improvement comparable with NB-UVB in inducing skin repigmentation [284]. In parallel, one study by Grimes reported that combination therapy involving the topical application of bimatoprost and mometasone resulted in greater repigmentation than either topical agent alone [285]. This effect may be attributed to the ability of PGs to stimulate human melanocyte dendricity, increase their size and number, and enhance the transfer of melanosomes to keratinocytes [149].

**Figure 2:**
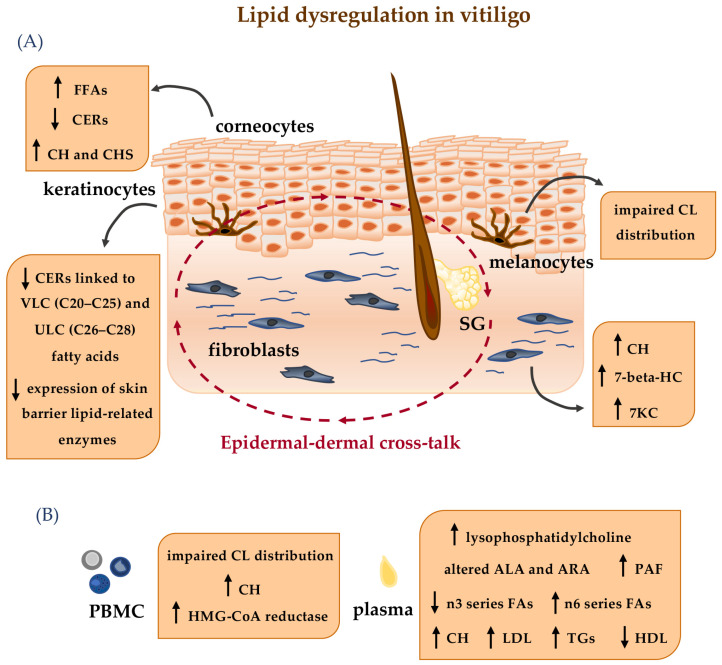
Schematic overview of lipid-related dysregulation highlighted in the cutaneous lipidome (**A**) and at systemic level (**B**) in vitiligo. FFAs: free fatty acids; CERs: ceramides; CH: cholesterol; CHS: cholesterol sulfate; VLC: very long chain; ULC: ultra long chain; CL: cardiolipin; HMG-CoA reductase: 3-Hydroxy-3-methylglutaryl coenzyme A reductase; ALA: alpha-linolenic acid; PAF: platelet-activating factor; ARA: arachidonic acid; FAs: fatty acids; TGs: triglycerides; LDL: low-density lipoprotein; HDL: high-density lipoprotein; 7KC: 7-ketocholesterol; 7-beta-HC: 7 beta-hydroxycholesterol; SG: sebaceous gland. The arrows in the Figure indicate the following: **↑** increased level of the indicated parameters; **↓** decreased level of the indicated parameters.

Targeting lipid pathways has been considered a potential approach for vitiligo. Treatment with the HMG-CoA reductase inhibitor simvastatin, used as a cholesterol-lowering agent, has shown promise. In vitro studies on human melanocytes have demonstrated its antioxidant action through the upregulation of nuclear erythroid 2-related factor (Nrf2) [286]. Furthermore, simvastatin has been demonstrated to be effective in reducing the extension and reversing the depigmentation in a mouse model of vitiligo [287]. However, despite a case of vitiligo regression being firstly reported in a patient treated with high doses of simvastatin [288], subsequent clinical studies have produced varying results. Some investigations have demonstrated the clinical ineffectiveness of oral or topical treatment, while another has described the successful control of disease activity in patients with dyslipidaemia (Figure 2) [289,290,291,292,293].

## 7. Conclusions and Perspectives

The advancement of lipidomics has led to significant progress in dermatological research. Indeed, skin lipidomics can provide qualitative and quantitative information on hundreds of biologically relevant lipid species with different properties and activities from a single skin sample. This makes it a valuable tool for translational studies on the role of lipids in skin health and disease and for identifying biological markers associated with inflammatory skin diseases.

However, despite the documented role of lipid mediators in melanogenesis and skin hyperpigmentary or hypopigmentary disorders, few studies have used a lipidomic approach to identify a lipid panel that could be used to diagnose and monitor skin diseases characterized by changes in pigmentation.

In the coming years, integrating lipidomics with other omics strategies, such as metabolomics, could overcome limitations in current studies. These limitations include small sample sizes, heterogeneous patient cohorts, and variability in analytical techniques. This integration could greatly improve the ability to study how alterations in skin surface lipids influence physiological pigmentation and diseases associated with increased or decreased skin pigmentation.

## Figures and Tables

**Table 1:** Lipid categories and their physiological effect on skin pigmentation, along with the corresponding references.

Lipid Category	Structure	Class and Subclasses	Physiological Effect on Skin Pigmentation	References
Fatty Acyls	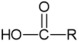	R = Alkyl chain of 4 to 28 carbons (usually even), with varying numbers of double bonds: 0: Saturated fatty acid 1: Monounsaturated fatty acid (MUFA) 2: Polyunsaturated fatty acid (PUFA)	Unsaturated fatty acids -inhibition of melanin synthesis -regulation of tyrosinase degradation Saturated fatty acids -tyrosinase stabilization -promotion of melanosome maturation	[129,130,131,132] [130]
Glycerophospholipids	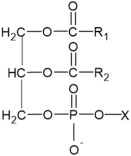	X= Choline: Phosphatidylcholine (PC) Ethanolamine: Phosphatidylethanolamine (PE) Inositol: Phosphatidylinositol (PI) Serine: Phosphatidylserine (PS)	-involvement in metabolic changes associated with pigmentation	[136,137,138]
Glycerolipids	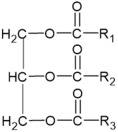	N of esterified acids= 1: Monoacylglycerol (MG) 2: Diacylglycerol (DG) 3: Triacylglycerol (TG)	-involvement in metabolic changes associated with pigmentation	[132,133,134,135]
Sphingolipids	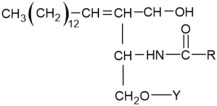 Sphingosine 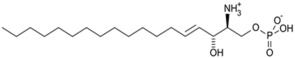 Sphingosine-1-P	Y= H: Ceramides Fatty acid: Sphingomyelin (SM)	-inhibition of pro-melanogenesis related signaling pathway -inhibition of tyrosinase activity	[158,159,160,161,162,163]
Sterols	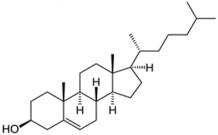 Cholesterol 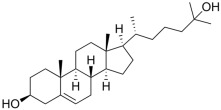 25-hydroxycholesterol		-induction of pro-melanogenesis related signaling pathway -inhibition of tyrosinase proteosomal degradation -induction of tyrosinase proteosomal degradation	[143,144] [145]
Prostaglandins	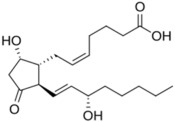 PGD_2_ 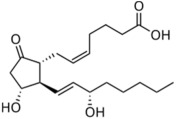 PGE_2_ 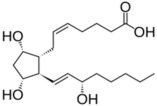 PGF_2α_		-induction of pro-melanogenesis related signaling pathway -induction of melanin synthesis -promotion of dendricity	[147,148,149]
Leukotrienes	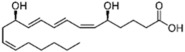 LTB4 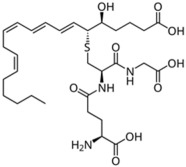 LTC4 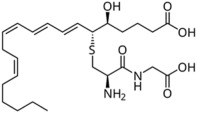 LTD4		-induction of tyrosinase expression -promotion of dendricity	[152]
Tromboxanes	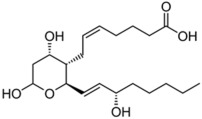 TBXB2		-induction of tyrosinase expression -promotion of dendricity	[152]
Endocannabinoids	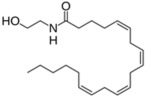 Anandamide or N-arachidonoylethanolamine (AEA) 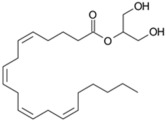 2-Arachidonoylglycerol (2-AG)		-induction of melanin synthesis -induction of pro-melanogenesis related signaling pathway	[172,173]

## Data Availability

No new data were created or analyzed in this study.
